# Biosecurity in Emerging Life Sciences Technologies, a Canadian Public Health Perspective

**DOI:** 10.3389/fpubh.2014.00198

**Published:** 2014-10-29

**Authors:** Kirsten X. Jacobsen, Kirsten Mattison, Marianne Heisz, Sandra Fry

**Affiliations:** ^1^Centre for Biosecurity, Public Health Agency of Canada, Ottawa, ON, Canada

**Keywords:** biosafety, biosecurity, pathogen, toxin, laboratory, dual use, risk management

Driven by innovation, science and technology are continually evolving. Over the past several years, the global scientific community and the world have had the opportunity to see firsthand the significant strides that have been made in the area of life science research, and the corresponding ethical, safety, and security questions that arise as a result of this work. The idea that well-intended research could be used for nefarious purposes is not new. The “dual-use” potential of advancing technologies has driven the dialog in a variety of sectors, including biological, chemical, and nuclear. In Canada, the Public Health Agency of Canada (PHAC) administers the *Human Pathogens and Toxins Act* (HPTA), the principle legislative tool overseeing the biosafety and biosecurity of activities involving human pathogens and toxins in Canada.

The HPTA currently requires all persons conducting controlled activities (possessing, handling, using, producing, storing, permitting access to, transferring, importing, exporting, releasing, or otherwise abandoning) with human pathogens and toxins to take all reasonable precautions to protect the health and safety of the public. Proposed regulations (*Human Pathogens and Toxins Regulations –* HPTR) to support full implementation of the HPTA (in 2015) were published online for public consultation until September 4, 2014 (http://www.gazette.gc.ca/rp-pr/p1/2014/2014-06-21/html/reg2-eng.php) and will be made final over the coming year. The HPTA framework has been developed through extensive consultation with regulated parties in order to keep the public safe and secure, while not inhibiting responsible scientific innovation and critical outbreak response activities. The key elements of the proposed framework are outlined here.

## A Proposed Licensing Regime

Socially responsible scientific innovation requires that the research community feels responsible for the outcomes of their research. Internationally, there are numerous approaches to establishing national biosafety and biosecurity accountability systems. The licensing regime that is proposed under the HPTA framework would marry these two perspectives by providing greater freedom to internally assess and manage risks with a corresponding increase in accountability for safe and responsible practices.

Under the proposed HPTR, a licensing scheme would be established for facilities conducting controlled activities with human pathogens and toxins. This risk-based scheme would impose more stringent biosafety and biosecurity requirements based on the inherent risks of the agents being handled and the nature of the activities being undertaken. Five elements of the proposed HPTA framework that are particularly relevant for the oversight of research with the potential for dual use and would be facilitated under the licensing scheme are as follows.

### Enhancing internal accountability systems

Certain stakeholder populations have risk management and oversight practices in place to support core business functions, such as quality control of their products, which also mitigate many biosafety risks. In other populations, institutional oversight can be highly variable. The proposed HPTR would require facilities conducting scientific research to submit information on how their facility administratively manages and controls biosafety and biosecurity risks, including information on roles and responsibilities of key biosafety personnel or committees. This is intended to enhance local oversight over pathogen research, the foundation of a “systemic” safety regime.

### Biological safety officers

Under an HPTA license, a qualified biological safety officer (BSO) would be designated for each institution (licensed entity) and this individual would have a number of duties and powers, including

- Verifying the accuracy and completeness of license applications.
- Communicating with PHAC on behalf of the institution as appropriate and necessary.
- Promoting and monitoring compliance.
- Assisting in the development and maintenance of the institution’s standard operating procedures related to biosafety and biosecurity and their biosafety manual.
- Assisting in internal investigations.
- Accessing all records necessary to carry out their functions.

The BSO would be a powerful resource for both the license-holder and PHAC to help oversee biosafety and biosecurity within an institution.

### Security requirements for “security sensitive biological agents (SSBAs)”

The proposed HPTR would establish a prescribed list of pathogens, including a subset of Risk Group 3 or 4 pathogens and prescribed toxins that are on the Australia Group common controls list (http://www.australiagroup.net/en/human_animal_pathogens.html). The proposed framework would require anyone with access to SSBAs to receive a security clearance, unless accompanied and supervised or exempted under the HPTR. Work with SSBAs would also be subject to additional biosafety and biosecurity requirements in the *Canadian Biosafety Standard*.

### “Gain of function” reporting

The proposed HPTA framework would support institutional risk management by requiring notification for experiments that will increase the risks posed by a pathogen (e.g., increased pathogenicity or virulence).

### Reporting events of public health significance

The proposed HPTA framework would further require that license holders notify PHAC of events (incidents, accidents) involving human pathogens or toxins that have the potential to put the public at risk. This includes inadvertent release, inadvertent production (e.g., through synthetic biology), inadvertent possession, missing or stolen pathogens or toxins, and any exposure that has or may have caused disease. These reporting requirements allow PHAC to assist in investigations, identify biosafety and biosecurity issues, and follow-up on potential issues of public health concern. Most of these events will not require direct action on behalf of the agency, but will assist in the ongoing and open dialog between regulators and stakeholders.

## A Collaborative Approach to Biosafety and Biosecurity

While the proposed framework provides a range of compliance monitoring, verification, and enforcement tools, PHAC focuses heavily on compliance promotion. Through extensive outreach and engagement, PHAC provides opportunities for open communication between researchers and regulators, enhancing overall biosafety and biosecurity. For example:

- Onsite compliance promotion inspections, which provide an opportunity for stakeholders to ask questions, receive input and recommendations for improvements, and gain confidence in their biosafety program.
- Promoting collaborative biosafety environments, for example, by assisting in the establishment of a Canadian University BSO Network and supporting conferences on Biosafety.
- Maintaining an active presence in the biosafety community, for example, at conferences, competitions (e.g., international Genetically Engineered Machines competition), and within academic institutions (e.g., assisting institutional biosafety committees).
- Stakeholder engagement in the development of the HPTA framework through consultations and expert working groups. In addition, an external Advisory Committee will be established under the HPTA to advise on the risks associate with human pathogens and toxins.
- Online training through the PHAC learning portal on topics such as biosafety principles, risk assessment, and dual use (http://www.publichealth.gc.ca/training).
- Pathogen Safety Data Sheets that describe the hazardous properties of a human pathogen and recommendations for work involving these agents in a laboratory setting (http://www.phac-aspc.gc.ca/lab-bio/res/psds-ftss/index-eng.php).
- Assisting with local risk assessments, site-specific risk assessments that consider not only the pathogen but also the specific activity being undertaken.
- Publishing Biosafety Advisories, Notifications, and Directives that communicate critical biosafety information to stakeholders, such as the recently updated advisory on influenza A/H7N9 (http://www.phac-aspc.gc.ca/lab-bio/res/index-eng.php).
- Comprehensive standards and robust guidelines to help stakeholders understand the biosafety and biosecurity requirements for working with pathogens and toxins (http://canadianbiosafetystandards.collaboration.gc.ca/index-eng.php).

PHAC has initiated a dialog with other Federal departments and agencies that have an interest in emerging life sciences and dual-use research. Together, we are examining where programs are robust, and where there are opportunities to improve oversight at all levels: among federal regulators, manufacturers, and distributors of enabling technologies, industry, researcher, the public, and anyone else with a stake in this complex issue. These conversations are happening in parallel around the world, providing further opportunities to look at the global context.

## Establishing a Culture of Responsibility

### Planning for success

For years, researchers have been trying to understand whether and how influenza A/H5N1 could become human-to-human transmissible by aerosols. A wide variety of approaches have been employed, but it was only when Drs. Kawaoka and Fouchier obtained relative success (1, 2) that the international community engaged in heated debate.

In 2006, when the National Institutes of Health recommended research on influenza viruses, including influenza virus transmission [National Institute of Allergy and Infectious Diseases (NIAID); (3)], there was an opportunity to initiate a dialog on the possible risks of such research. Had the international scientific community started to discuss the possible dual-use implications of actually succeeding in creating a mammalian transmissible highly pathogenic avian influenza virus in the laboratory, at the very least, we would have been more prepared when it occurred in 2011 (1, 2). These and other examples tell us that, in the global arena, we have a way to go in planning for success from the perspective of biosafety and biosecurity, which may include early involvement of regulators and oversight bodies in the planning stages. As science and technology continue to advance, the challenges associated with “planning for success” will increase exponentially, and policy makers will need to determine how to adjust, for example, to a reality where one can create an entire biological system that has never been seen before.

### A culture of responsibility

In a 2011 report on strengthening the culture of responsibility in the context of biosecurity [National Science Advisory Board for Biosecurity (NSABB); (4)], the NSABB writes that “knowledge is rarely, if ever, neutral.” Information of almost any type can be used for both positive and negative applications and thus, determining what knowledge presents the greatest risk for dual use is not only difficult but also highly subjective. Within the realm of life sciences research, external bodies, such as federal regulators, can play an important role in education and enforcing accountability. A culture of responsibility, however, cannot be legislated, but it can be cultivated. In this scenario, everyone with an interest in the great potential benefits and possible risks associated with cutting edge life sciences research has a role to play.

The NSABB report (4) details the role researchers have in understanding the possible implications and applications of their work, in championing good research practices, and in taking ownership of their own responsibility by holding themselves and their peers accountable. This philosophy could equally be extended beyond researchers to the wider community: to institutions, to the manufacturers and distributors of enabling technologies, to professional associations, and even to civil society and the public.

Social responsibility is much larger than individual researchers. It is widely accepted that working in silos is disruptive to institutional alignment and collaboration. Silo mentality has added disadvantage of diffusing safety, security, and ethical practices, leading to redundancies and possible gaps. There is a significant advantage to systemizing safety and security practices – increasing both the responsibility and the accountability of institutions for the oversight and the outcomes of the research done within. In Canada, the proposed framework would place responsibility on research institutions for administrative oversight, permitting a tailored approach to risk management to suit the unique research environment. Establishing biosafety and biosecurity programs such that risk management occurs in a collaborative and integrated environment is expected to increase the likelihood that the necessary conversations take place long before research with potential for dual use is underway. This would then inform the wider dialog on the ethics, safety, and security issues related to emerging biological sciences.

### Risk–benefit analysis

In recent examples of research with the potential for dual use, international discussions focused on the risks of publication, with less emphasis on the need for systematic, scientific, evidence-based risk–benefit analysis in such research (5, 6). In the absence of concrete data, as is the case with emerging technologies, this risk–benefit analysis may be largely hypothetical. The risks of potential misuse (accidental or intentional) are weighed against the assumed potential benefits of scientific innovation. Within the scientific community, there is growing debate over the latter with respect to “gain-of-function” flu research. On October 16, 2013, a letter was sent to the President of the European Commission on behalf of the European Society for Virology (ESV), cautioning against prohibiting dual-use research because of the potential benefits (7). Two months later, a letter in response to the ESV appeal was sent to President Barroso on behalf of the Foundation for Vaccine Research, challenging many of the reputed benefits (8).

This underscores the need to take a critical look at the real benefits of research with potential for dual use and, in some way, measure them against the real risks. This will be very difficult as, in almost all cases, the “real” risks and benefits have not yet been realized, and there may be significant division within the scientific community on both counts. This is perhaps the area of discussion in which it is most important to involve all sectors, including the public, as “risk” and “benefit” are both highly influenced by perception. A qualitative risk–benefit analysis framework for assessing research with dual-use potential, if possible, would be the most decisive tool for asking the hardest and most important questions we currently face: “what happens if we do not do this research” but also “what happens if we do.”

## Conflict of Interest Statement

The authors declare that the research was conducted in the absence of any commercial or financial relationships that could be construed as a potential conflict of interest.

## References

[1] Herfst S Schrauwen EJ Linster M Chutinimitkul S de Wit E Munster VJ Airborne transmission of influenza A/H5N1 virus between ferrets. Science (2012) 336(6088):1534–41. 10.1126/science.1213362 22723413 PMC4810786

[2] Imai M Watanabe T Hatta M Das SC Ozawa M Shinya K Experimental adaptation of an influenza H5 HA confers respiratory droplet transmission to a reassortant H5 HA/H1N1 virus in ferrets. Nature (2013) 486(7403):420–8. 10.1038/nature10831 22722205 PMC3388103

[3] National Institute of Allergy and Infectious Diseases (NIAID). Report of the Blue Ribbon Panel on Influenza Research. National Institutes of Health (2006). Available from: http://www.niaid.nih.gov/topics/flu/documents/influenzablueribbonpanel2006.pdf

[4] National Science Advisory Board for Biosecurity (NSABB). Guidance for Enhancing Personnel Reliability and Strengthening the Culture of Responsibility. (2011). Available from: http://osp.od.nih.gov/sites/default/files/resources/CRWG_Report_final.pdf

[5] van Aken J. When risk outweighs benefit. Dual-use research needs a scientifically sound risk-benefit analysis and legally binding biosecurity measures. EMBO Rep (2006) 7:s10–3. 10.1038/sj.embor.7400726 16819441 PMC1490308

[6] Engel-Glatter S. Dual-use research and the H5N1 bird flu: is restricting publication the solution to biosecurity issues? Sci Public Policy (2014) 41:370–83. 10.1093/scipol/sct064

[7] Palù G. ESV Letter on Gain of Function Research in Virology. (2013). Available from: http://www.eusv.eu/pdf/ESV%20letter%20on%20Gain%20of%20function_GOF_research%20in%20Virology.pdf

[8] Wain-Hobson S Weiss RA Hale P Gatell JM Hanekom W Le Gall S Response to Letter by the European Society for Virology on “Gain-of-Function” Influenza Research and Proposal to Organize a Scientific Briefing for the European Commission and Conduct a Comprehensive Risk-Benefit Assessment. (2013). Available from: http://news.sciencemag.org/sites/default/files/media/Letter%20to%20Barroso_0.pdf

